# CRISPR-Cas9-AAV versus lentivector transduction for genome modification of X-linked severe combined immunodeficiency hematopoietic stem cells

**DOI:** 10.3389/fimmu.2022.1067417

**Published:** 2023-01-04

**Authors:** Julie Brault, Taylor Liu, Siyuan Liu, Amanda Lawson, Uimook Choi, Nikita Kozhushko, Vera Bzhilyanskaya, Mara Pavel-Dinu, Ronald J. Meis, Michael A. Eckhaus, Sandra S. Burkett, Marita Bosticardo, Benjamin P. Kleinstiver, Luigi D. Notarangelo, Cicera R. Lazzarotto, Shengdar Q. Tsai, Xiaolin Wu, Gary A. Dahl, Matthew H. Porteus, Harry L. Malech, Suk See De Ravin

**Affiliations:** ^1^ Laboratory of Clinical Immunology and Microbiology, National Institute of Allergy and Infectious Diseases (NIAID), National Institutes of Health (NIH), Bethesda, MD, United States; ^2^ Cancer Research Technology Program, Leidos Biomedical Research Inc., Frederick, MD, United States; ^3^ Department of Pediatrics, Division of Stem Cell Transplantation and Regenerative Medicine, Stanford University, Palo Alto, CA, United States; ^4^ Cellscript LLC Inc., Madison, WI, United States; ^5^ Division of Veterinary Resources, Office of Research Services, National Institutes of Health, Bethesda, MD, United States; ^6^ Molecular Cytogenetic Core Facility, Center for Cancer Research, National Cancer Institute, National Institutes of Health, Frederick, MD, United States; ^7^ Center for Genomic Medicine and Department of Pathology, Massachusetts General Hospital, Boston, MA, United States; ^8^ Department of Pathology, Harvard Medical School, Boston, MA, United States; ^9^ Department of Hematology, St. Jude Children’s Research Hospital, Memphis, TN, United States

**Keywords:** preclinical studies, CRISPR-Cas9, AAV6, XSCID, lentivector

## Abstract

**Introduction:**

*Ex vivo* gene therapy for treatment of Inborn errors of Immunity (IEIs) have demonstrated significant clinical benefit in multiple Phase I/II clinical trials. Current approaches rely on engineered retroviral vectors to randomly integrate copy(s) of gene-of-interest in autologous hematopoietic stem/progenitor cells (HSPCs) genome permanently to provide gene function in transduced HSPCs and their progenies. To circumvent concerns related to potential genotoxicities due to the random vector integrations in HSPCs, targeted correction with CRISPR-Cas9-based genome editing offers improved precision for functional correction of multiple IEIs.

**Methods:**

We compare the two approaches for integration of *IL2RG* transgene for functional correction of HSPCs from patients with X-linked Severe Combined Immunodeficiency (SCID-X1 or XSCID); delivery *via* current clinical lentivector (LV)-*IL2RG* versus targeted insertion (TI) of *IL2RG via* homology-directed repair (HDR) when using an adeno-associated virus (AAV)-*IL2RG* donor following double-strand DNA break at the endogenous *IL2RG* locus.

**Results and discussion:**

*In vitro* differentiation of LV- or TI-treated XSCID HSPCs similarly overcome differentiation block into Pre-T-I and Pre-T-II lymphocytes but we observed significantly superior development of NK cells when corrected by TI (40.7% versus 4.1%, p = 0.0099). Transplants into immunodeficient mice demonstrated robust engraftment (8.1% and 23.3% in bone marrow) for LV- and TI-*IL2RG* HSPCs with efficient T cell development following TI-*IL2RG* in all four patients’ HSPCs. Extensive specificity analysis of CRISPR-Cas9 editing with rhAmpSeq covering 82 predicted off-target sites found no evidence of indels in edited cells before (*in vitro*) or following transplant, in stark contrast to LV’s non-targeted vector integration sites. Together, the improved efficiency and safety of *IL2RG* correction *via* CRISPR-Cas9-based TI approach provides a strong rationale for a clinical trial for treatment of XSCID patients.

## Introduction

X-linked severe combined immunodeficiency (SCID-X1 or XSCID) is caused by mutations in *IL2RG* gene which encodes the common cytokine gamma chain (γc) subunit shared by multiple cytokine receptors (interleukin (IL)-2, 4, 7, 9, 15 and 21). These cytokines are necessary for the development, differentiation, and function of T, B and NK cells, defects of which result in profound immune deficiency and risks of death in infancy if untreated.

Allogeneic hematopoietic stem/progenitor cell transplant (HSCT) potentially cures SCID; the best outcomes achieved when performed early (<3.5months of age), infection free, with HLA-matched, related donors (1). Transplants from haploidentical parents without myeloid conditioning is life-saving, but often results in engraftment of only T cell progenitors. To address donor availability and immune-incompatibility issues, *ex vivo* CD34 stem cell gene therapy was developed (2). Due to insertional mutagenesis events associated with the initial autologous HSCT gene therapy using γRV (3, 4), vectors for clinical gene therapy have evolved to self-inactivating γRV (5, 6) or lentiviral (7, 8) vectors that deliver a codon-optimized *IL2RG* driven by an exogenous promoter.

Self-inactivating lentivector gene therapy with myeloid conditioning have provided significant benefit to SCID-X1 patients (7, 8). Improvement of immunity in the older patients with XSCID was delayed and potentially related to the relatively weak exogenous EF1a promoter. In addition to myelodysplastic events recently reported in LV-treated cerebral adrenoleukodystrophy patients (bluebird bio; Servick K. Gene therapy clinical trial halted as cancer risk surfaces. Science News. 2021. https://www.science.org/content/article/gene-therapy-clinical-trial-halted-cancer-risk-surfaces), aberrant vector-gene fusion transcripts causing clonal expansions were reported in LV XSCID and β-thalassaemia patients (9–11). The long-term impact of random vector insertion and potential for oncogenesis remains a long-term concern.

CRISPR-Cas9 approach of targeted *IL2RG* insertion previously reported up to 20% targeted integration frequencies in long-term HSCs efficient rates of correction and no detectable off-target activity (12), thus potentially provide a safer genotoxicity profile than random insertions. However, *ex vivo* targeted genome editing of HSPCs generally requires delivery of gene-editing (GE) components by electroporation, relies on the induction of double strand DNA cleavages, and exposure to recombinant adeno-associated virus (AAV) for delivery of corrective donor. These processes induce DNA damage responses that potentially adversely affect cell fitness and engraftment of edited HSPCs.

In this study, we report further optimization of the CRISPR-Cas9-AAV *IL2RG* method to compare its efficacy with clinical lentivector (12, 13). The engraftment rates of GE and LV-transduced cells were comparable, and highly efficient levels of TI (63.6 ± 11.4% and up to 84.7% *in vitro*) were maintained 16-18 weeks after transplant into immunodeficient mice (52.6 ± 16.5% *in vivo* in the bone marrow).

Importantly, the *in vitro* differentiation of gene corrected XSCID HSPCs into NK cells was superior with CRISPR-Cas9 edited cells. Our investigation of genomic integrity and preclinical studies further demonstrated the safety of this approach and provide the information and rationale for the translation of the CRISPR-Cas9-targeted *IL2RG* insertion approach to treat XSCID patients.

## Material and methods

### Human samples

Cells from healthy volunteer donor (HD) and XSCID patients were obtained from the National Institutes of Health Department of Transfusion Medicine, Center for Cellular Engineering, after written informed consent under auspices of National Institute of Allergy and Infectious Diseases (NIAID) institutional review board (approved protocols NIAID IRB 05-I-0213 and 94-I-0073). The conduct of these studies conforms to the Declaration of Helsinki protocols and all US federal regulations required for protection of human subjects. Subjects underwent leukapheresis after CD34^+^ HSPC mobilization with G-CSF (15 mg/kg daily) for 5 days and plerixafor at 12 hours before blood collection. Purified CD34^+^ HSPCs were cryopreserved immediately after separation.

### Design of single-guide RNA and rAAV6-*IL2RG*

*Streptococcus pyogenes* Cas9 (SpCas9), single guide RNAs (sgRNAs) containing 20 nucleotide (20 nt) spacers or those with truncated 19 nt spacers targeting Exon1 of *IL2RG* locus were designed as previously described (12, 14)

The recombinant adeno-associated virus serotype 6 (rAAV6)-*IL2RG* donor plasmid was designed as previously described (12), with codon-optimization of *IL2RG* cDNA, WPRE enhancer sequence and β-thalassaemia-globin polyA tail, flanked by 0.4-kb homology arms. This plasmid was used for large-scale production of rAAV6-*IL2RG* donor (Vigene Biosciences, Inc, Rockville, MD).

### Gene correction of CD34^+^ HSPCs

After thawing cryopreserved CD34^+^ HSPCs, cells were pre-stimulated for 48hours in StemSpanII medium (StemCell Technologies) or X-Vivo™ 10 (Lonza) for gene editing and lentiviral transduction respectively, supplemented with 100 ng/mL Stem cell factor (SCF), Flt3-ligand (Flt3-L) and Thrombopoietin (TPO) (Peprotech), at a seeding density of 0.5x10^6^ cells/mL.

Gene editing was performed by electroporation with sgRNA (Synthego), SpCas9 mRNA, inhibitor of 53 binding protein 1 (i53) mRNA, human truncated dominant p53 inhibitor (GSE CS-56) mRNA (CellScript LLC) and 2% glycerol as previously described (15–17) followed by transduction with AAV-*IL2RG* donor template. To evaluate the kinetic of expression of i53 and GSE CS-56 after electroporation and gene editing, tagged version of i53 and GSE CS-56 (i53-FLAG and GSE-HIS) instead of i53 or GSE CS-56 alone mRNAs were produced using the same process (CellScript LLC).

Lentivirus transduction was performed 2 days after thawing and culture with the clinical lentivector Cl20-i4-EF1a-*IL2RG* using the enhanced transduction procedure with LentiBoost (Sirion Biotech) and 16,16-dimethyl-prostaglandin E2 (Cayman Chemical) as previously described (11).

Cells were maintained in the same medium post-treatment. Then, after 48h, cells were harvested and counted by hemocytometer with trypan blue to determine viability before downstream analysis: phenotype, CFU assay, T or NK cell differentiation, or cryopreservation for transplant studies.

### *In vitro* T and NK cell differentiation

CD34^+^ HSPCs were differentiated *in vitro* into T cells using a 3D artificial thymic organoid (ATO) system (11, 15, 18, 19) and into NK cells as previously described (15).

### Transplantation studies

The use of triple transgenic mice NOD.Cg-Prkdc*^scid^* Il2rg*^tm1Wjl^* Tg (NSG-SGM3 or NSGS) expressing human IL3, GM-CSF and SCF immunodeficient mice (20) (Stock No: 013062; The Jackson Laboratory) for transplantation studies was approved by NIAID Institutional Animal Care and Use Committee under the animal use protocol LCIM 1E. The conduct of these studies conforms to AAALAC International guidelines and all US federal regulations required for protection of research animals. Newborn mice (0-4 days old) were irradiated at 100 cGy and intrahepatically injected with 1-1.5x10^6^ viable CD34^+^ cells per mouse for the primary transplant and 2-3x10^6^ viable human CD45^+^ cells per mouse for the secondary transplant. Peripheral blood (PB) from tail vein was analyzed at weeks 12 and 16-18 after transplantation. At 16-18 weeks, bone marrow (BM), spleen and thymus were harvested for analysis of engraftment, sorting or gDNA extraction as indicated.

### Flow cytometry

Briefly, cells were washed in FACS buffer (PBS with 0.1% bovine serum albumin) and centrifuged for 5 minutes at 1800 rpm. For cell surface staining, cells were incubated for 30 minutes in the dark with antibodies and washed in FACS buffer. The list of antibodies is provided in the **Supplemental Table 1**.

Intracellular pSTAT5 assay was performed as previously described (12, 21) with minor changes. Briefly, cells harvested from the spleen were pelleted and resuspended in PBS, then left unstimulated or stimulated with 10,000 U/mL rhIL2 and 10 ng/mL rhIL15 (NK cells) or rhIL7 (T cells) for 15 min at 37°C. After washing and fixation with PFA 4% for 10 min at 37°C, cells were permeabilized by incubation with ice-cold methanol (added dropwise) on ice for 30 min. Cells were washed with PBS with 1% fetal bovine serum (FBS) and incubated for 1h at room temperature with APC-conjugated CD45 (BD Biosciences), APC-Cy7-conjugated CD3 (BioLegend) and AF488-conjugated pSTAT5 (BD Biosciences). Cells were then washed and analyzed.

Flow cytometry analysis was performed using a BD Canto or a BD Fortessa flow cytometer, DIVA software (BD Biosciences) and FlowJo analysis software v10.6.3 (Tree Star).

### Cell sorting

Spleen samples were washed and surface staining for cell sorting was performed by pelleting cells and resuspending in 100 µl of FACS buffer for 25 min at 4 °C in the dark with the following antibodies: PE-conjugated CD45 (BD Biosciences), APC-conjugated CD33 (BD Bioscience), APC-Cy7-conjugated CD3 (BioLegend), BV421-conjugated CD19 (BioLegend) and FITC-conjugated CD56 (BioLegend). Cells were washed once in FACS buffer and filtered through a 40-µm filter before resuspension in HBSS with 5% FBS with 7-AAD staining in order to exclude dead cells. Cells were sorted using a FACS-Aria Illu cell sorter (BD Biosciences) at the Flow Cytometry Section (Research Technologies Branch, DIR, National Institute of Allergy and Infectious Diseases, NIH).

### Quantification of IgG in the serum of transplanted mice

Serum was aliquoted after blood collection at week 16-18 post-transplant. Titration of human IgG was performed using IgG (Total) Human ELISA Kit (BMS2091; ThermoFisher Scientific) according to manufacturer’s instructions.

### Quantification of on-target indel activity, TI and vector copy number

gDNA extraction was performed on HSPCs CD34^+^ cells (5 days post-GE and 14 days post-LV transduction), NK or T cells using QIAamp^®^ DNA mini kit (QIAGEN) according to manufacturer’s instructions.

Insertion or deletion mutations (indels) at *IL2RG* exon 1 were quantified using TIDE (Tracking of Indels by Decomposition; https://tide.deskgen.com/) (22) after PCR amplification (primers Fwd 5'-GAAGGTAATGATTTAGAGGAGAAGGTG and Rev 5'-AGCCTAGGCAACATAGTGAGACCCTG) and Sanger sequencing (CCR Genomics Core, NCI, NIH).

TI of *IL2RG* cDNA after gene editing was quantified by digital droplet PCR (ddPCR) using one primer located upstream of the LHA (5’-GGTGACCAAGTCAAGGAAGAG), the second primer inside the cDNA (5’- GGTGAGGATGGTGGTATTCAAG) and a probe (5’- TGCTGTTTCTCCAACTCCCTCTGC).

For the measurement of VCN, vector-specific primers and probes were used as follows: HIV forward, 5′-CTGTTGTGTGACTCTGGTAACT-3′; HIV reverse, 5′-TTCGCTTTCAAGTCCCTGTT-3′; and HIV probe, 5′-/56-FAM/AAATCTCTA/ZEN/GCAGTGGCGCCCG/3IABkFQ/-3′.

Control primers were used to amplify the myocardin-like protein 2 (MKL2) gene also located on chromosome X (forward, 5′-AGATCAGAAGGGTGAGAAGAATG-3′; reverse, 5′-GGATGGTCTGGTAGTTGTAGTG-3′; and probe, 5′-/56-HEX/TGTTCCTGC/ZEN/AACTGCAGATCCTGA/3IABkFQ/-3′).

### Identification and quantification of off-targets

Identification of off target sites was performed using Circularization for high throughput analysis of nuclease genome-wide effects by sequencing (CHANGE-seq) on genomic DNA extracted from human CD34^+^ cells as previously described (23).

Then quantification of indel activity at off-target sites was performed using rhAmpSeq technology for ultradeep sequencing of 82 Off targets identified on CHANGE-seq (24).

### Karyotype and fluorescent *in situ* hybridization

XSCID CD34^+^ cells gene edited were prepared for chromosomal analysis and FISH as previously described. The metaphases were hybridized with the 24-color human SKY paint kit (FPRPR0028, ASI). Twelve metaphases were imaged for karyotype. Spectral images of the hybridized metaphases were acquired using Hyper Spectral Imaging system (Applied Spectral Imaging) mounted on top of an epi-fluorescence microscope (Imager Z2, Zeiss). Images were analyzed using HiSKY 8.2 acquisition software (Genasis, Applied Spectral Imaging). G-banding was simulated by electronic inversion of DAPI counterstaining. An average of 10–15 mitoses of comparable staining intensity and quality was examined per cell line and compared for chromosomal abnormality. The karyotype was determined by comparison to the standard ideogram of banding patterns for human chromosomes.

### Hematology, chemistry and pathology analysis

Blood was collected in tube without anticoagulant and serum separated immediately by centrifugation for chemistry or collected in EDTA tubes for hematology cell blood count (CBC). Both chemistry and hematology analyses were performed by the Department of Laboratory Medicine (DLM) (Veterinary Services, Clinical Center, NIH).

Necropsy was performed by Pathology Service of the Division of Veterinary Resources (ORS, OD, NIH) in order to investigate for signs of tumor/malignancy in animals.

### Statistical analysis

Results were presented as means ± standard deviation (SD). Statistical testing was performed using GraphPad Prism.

## Results

### Evaluation of cell fitness after gene editing or lentiviral transduction of HSPCs

**Figure 1:**
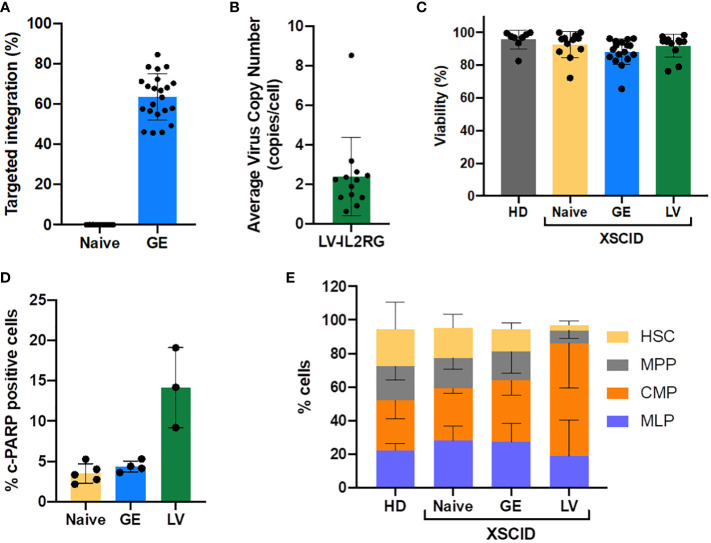
Efficacy of CRISPR-Cas9-AAV gene editing and LV-transduction and impact on the cell fitness. Frequency of TI of *IL2RG* after gene editing (GE) **(A)** and the average VCN after LV-transduction **(B)** into HD and XSCID patients CD34^+^ HSPCs, as determined by ddPCR. Each symbol depicts a sample, from 9-14 independent experiments, 6 individual patients. **(C)** Cell viability of CD34^+^ XSCID HSPCs measured by trypan blue exclusion at 2 days post GE or LV-transduction. **(D)** c-PARP1 expression measured by flow cytometry at 24h post-treatment of CD34^+^ XSCID HSPCs; n=3-5 independent experiments. **(E)** Repartition of CD34^+^ subpopulations at day2 post treatment; n=3-5 independent experiments (1 HD, 2 XSCID patients).

To assess potential damage to HSPCs from the genetic modifying process, we first compared the fitness of mobilized peripheral blood CD34^+^ HSPCs from 2 HD and 6 XSCID patients following CRISPR-Cas9-AAV GE or lentiviral transduction. Gene editing was performed by electroporating HSPCs with SpCas9 mRNA and sgRNAs with 20bp spacers (sg-20bp) in the presence of gene editing enhancers to favor HDR-mediated correction using i53 (16, 17) and dampen the p53-mediated DNA damage response induced after AAV transduction using GSE CS-56 (15). Cells were then immediately transduced with rAAV6-*IL2RG* HDR donor. Using these optimized gene editing conditions, TI rates measured by ddPCR reached 63.6%±11.4% in GE HSPCs (**Figure 1A**). In parallel, HSPCs transduced with the current clinical lentivector Cl20-i4-EF1a-*IL2RG* in presence of transduction enhancers (LentiBoost and dimethyl-prostaglandin E2) showed an average VCN of 2.4 copies/cell (**Figure 1B**).

For both approaches (GE and LV), cell viability at two days post-treatment was maintained at the level of HD and Naive (untreated) XSCID cells (**Figure 1C**). However, the expression of c-PARP1 measured at 24h post-treatment showed a higher percentage of cells undergoing apoptosis when transduced with LV compared to GE therapy (**Figure 1D**). In addition, the phenotype of HSPCs at 48h post-treatment was perturbed after LV-transduction with 2 to 3-fold reduction of HSC (CD34^+^CD38^-^CD45RA^-^CD90^+^) and multi-potential progenitors (MPP; CD34^+^CD38^-^CD45RA^-^CD90^-^) and increase of common myeloid progenitor (CMP; CD34^+^CD38^+^CD45RA^-^) population compared to HD, Naive and GE XSCID (**Figure 1E**). Finally, the hematopoietic potential evaluated *in vitro* using the colony-forming unit assay showed no significant reduction of the number of colonies for GE and LV-transduced HSPCs compared to Naïve XSCID HSPCs (**Supplemental Figure S1A**) and no abnormality in the cell cycle was detected (**Supplemental Figure S1B**).

### Quantification and reduction of off-target activity with sgRNAs targeting IL2RG locus

**Figure 2:**
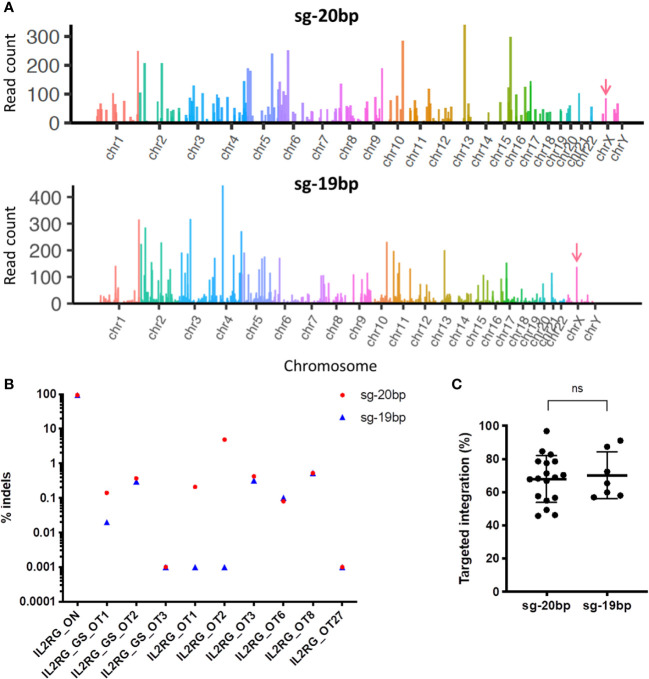
Comparison of off-target activity using sg-20bp versus truncated sg-19bp. **(A)** Manhattan plot for CHANGE-seq results performed using *IL2RG* sg-20bp or sg-19bp. **(B)** HTS results for on- and off-target editing in cells treated with *IL2RG* sg-20 and sg-19. **(C)** Percentage of TI of the *IL2RG* cDNA at the endogenous locus determined by ddPCR (6-12 independent experiments) ns, non-significant.

Double-strand DNA breaks (DSBs) at genomic sites other than the targeted locus result in insertions or deletions (Indels), or off-target (OT) sites. *In vitro* assays such as Circularization for High throughput Analysis of Nuclease Genome-wide Effects by sequencing (CHANGE-seq) assay (23) induce cleavage reactions using SpCas9 protein to identify potential DSBs. Using this approach, 686 potential OT sites were nominated (**Figure 2A**). However, after targeted High-Throughput Sequencing of top 10 CHANGE-seq ranked OT sites, only the predicted OT2 located in the intron of *MPZL1* gene showed 4-8% indel activity in GE HSPCs in presence of AAV-*IL2RG* donor and sg-20bp with background noise of ~2-3% (**Figure 2B**). This OT editing was reduced to background levels (0.001%) when using a truncated version of the same sgRNA, sg-19bp (**Figure 2B**). To evaluate a broader range of nominated OTs, 82 targets were assessed using a recently described rhAmpSeq (25). RhAmpSeq detected efficient on-target TI but none detected at the OT sites analyzed. We also confirmed that gene editing with sg-19bp resulted in similar efficiencies for TI than previously reported (12) (**Figure 2C**).

### Phenotypic and functional correction in T and NK cells after *in vitro* differentiation

**Figure 3:**
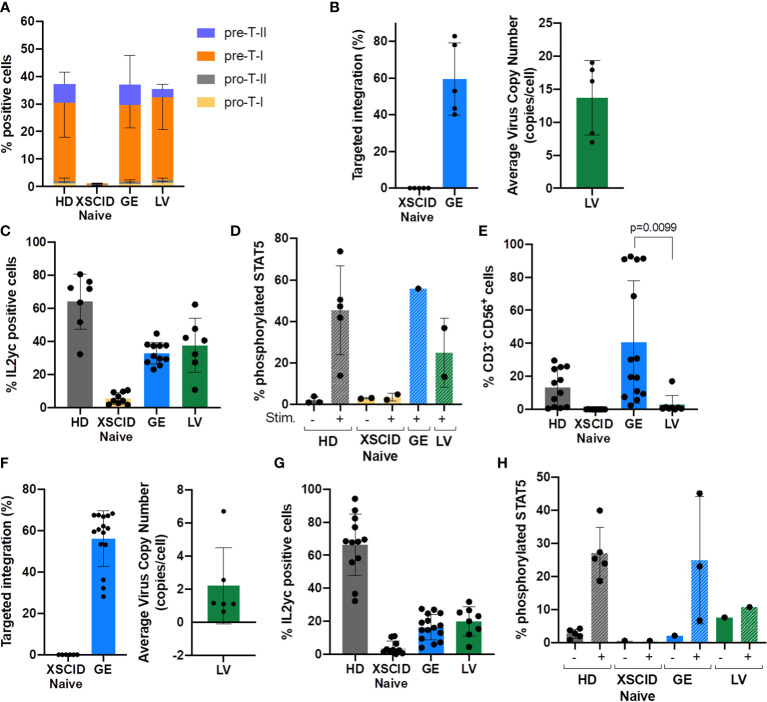
Evaluation of *in vitro* development of T and NK cells from GE- or LV-treated XSCID HSPCs and phenotypic/functional characterization. **(A)** Bar graph showing the relative proportion of CD34-derived T progenitors and precursor cells in each condition measured by flow cytometry at week 6 of *in vitro* differentiation (n = 9 independent experiments). **(B)** Percentage of TI and VCN quantified by ddPCR at week 6 of *in vitro* T cell differentiation (n = 5 independent experiments). **(C)** Percentage of T cells expressing IL2γc measured by flow cytometry at week 6 of *in vitro* differentiation (n = 7 independent experiments). **(D)** Percentage of T cells with phosphorylated STAT5 after 15min of stimulation with IL2/IL7 (+) or left unstimulated (-) (n = 1-4 independent experiments). **(E)** Bar graph showing the percentage of CD34-derived NK cells (CD3^-^CD56^+^) in each condition measured by flow cytometry at day 35 of *in vitro* NK differentiation (n = 12 independent experiments). **(F)** Percentage of TI and VCN quantified by ddPCR at day 35 of *in vitro* NK differentiation (n = 12 independent experiments). **(G)** Percentage of cells expressing IL2γc measured by flow cytometry in NK cells at day 35 of *in vitro* differentiation (n = 12 independent experiments). **(H)** Percentage of NK cells with phosphorylated STAT5 after 15min of stimulation with IL2/IL15 (+) or left unstimulated (-) (n = 3-5 independent experiments).

IL2γc-mediated signaling is essential for T and NK cell development, thus we evaluated the ability of GE- or LV-treated XSCID HSPCs to differentiate *in vitro* into either T cells or NK cells using respective *in vitro* differentiation approaches (**Figure 3**).

Serum-free 3D artificial thymic organoid (ATO) system is an excellent *in vitro* model for evaluating T cell differentiation from CD34^+^ HSPCs (15, 18, 19). The ATOs seeded with Naive XSCID HSPCs were blocked at the CD34^+^ Pro-T stage with very few that had progressed to pre-T-I stage (**Figure 3A**). In contrast, both GE- and LV-treated XSCID HSPCs overcame the blockade to progress to pre-T-I and pre-T-II precursors as observed in HD HSPCs (**Figure 3A**; **Supplemental Figure S2A**). Molecular analysis using ddPCR showed an average TI of 59.5% (range 40.16-78.02%) in GE samples and 13.68 VCN/cell in LV samples (**Figure 3B**). This is consistent with an increased IL2γc expression observed by flow cytometry in ATO-harvested cells (**Figure 3C**) and a restoration of the γc-mediated signaling as evidenced by STAT5 phosphorylation upon stimulation with IL2/IL7 (**Figure 3D**).

Of note, consistent with clinical observations, LV-transduced XSCID HSPCs failed to differentiate into NK cells (mean: 2.8%, range 0.5-17.0%) while GE XSCID HSPCs successfully differentiated into NK cells (mean: 40.7%, range 2.41-92.5%) (**Figure 3E**; **Supplemental Figure S2B**). The genomic correction showed an average TI of 63.6% (range 45.74-84.66%) in the NK cells differentiated from the GE XSCID HSPCs and 2.22 VCN/cell from the LV-transduced XSCID HSPCs (**Figure 3F**). Increase in IL2γc expression (**Figure 3G**) and phosphorylation of STAT5 after stimulation of the NK cells with IL2/IL15 (**Figure 3H**) confirmed phenotypic and functional correction.

### Transplants into immunodeficient mice

**Figure 4:**
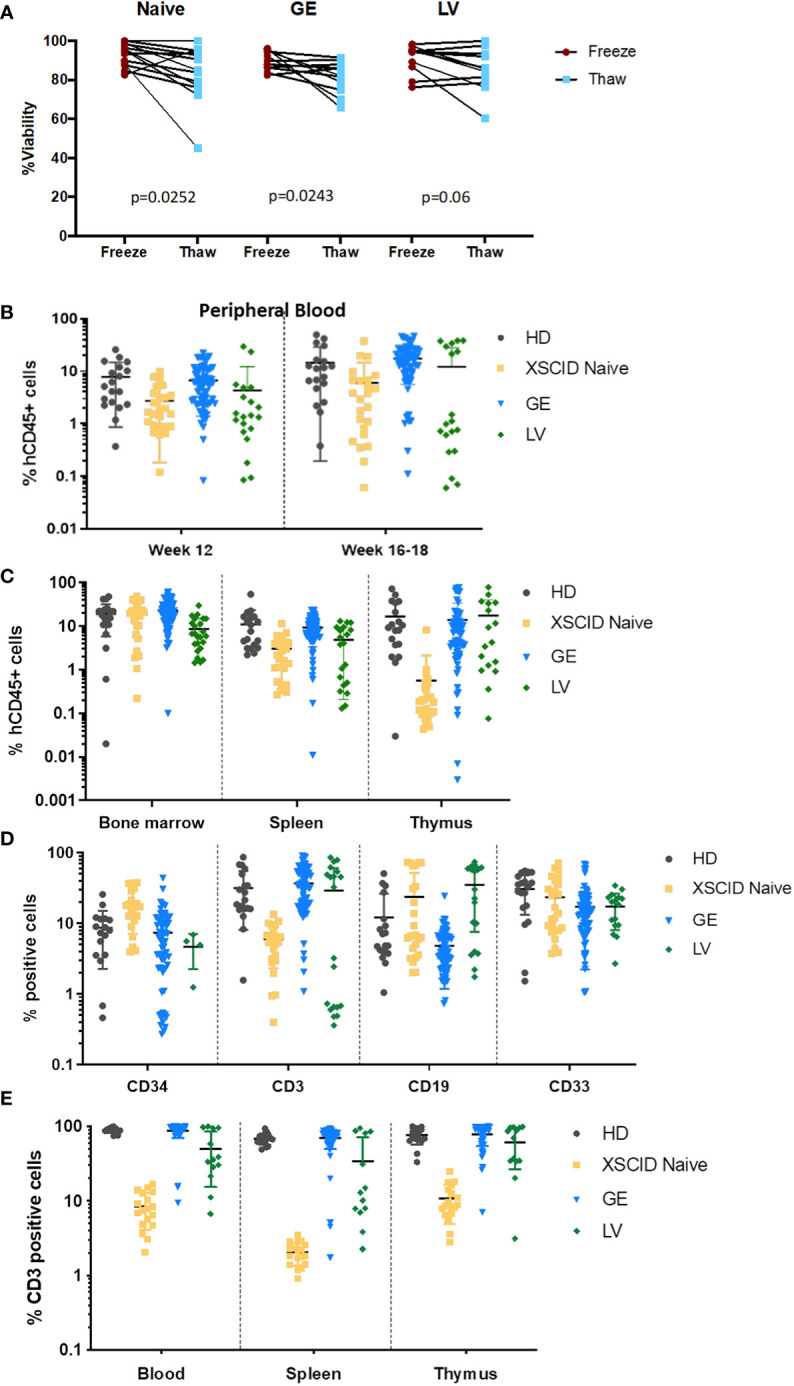
*In vivo* engraftment and T-cell development in immunodeficient mice transplanted with GE- and LV-treated XSCID HSPCs **(A)** Percentage of cell viability at freezing post processing and at thaw (second cycle freeze-thaw) following processing of XSCID HSPCs Naive and after GE or LV-transduction (n = 6-10 independent experiments). **(B)** Analysis of peripheral blood from mice transplanted with HSPCs from HD, XSCID Naive or GE- and LV-treated at weeks 12 and 16-18 for human engraftment (hCD45^+^). **(C)** Level of human CD45^+^ engraftment in mice bone marrow, spleen and thymus at 16-20 weeks after transplant with HD, XSCID Naïve, GE- and LV-treated HSPCs. **(D)** Percentages of human CD34^+^, myeloid (CD33^+^), B lymphoid (CD19^+^) and T lymphoid (CD3^+^) cells in the BM at week 16-18 post-transplant (gating on hCD45^+^). **(E)** Percentage of CD3^+^ T cells in the PB, spleen and thymus at week 16-20 post-transplant. HSPCs are from 2 HD and 4 XSCID patients. Data are showed as mean ± standard deviation. Each symbol indicates a single animal.

Cryopreservation of investigational medical products following genomic modification has been strongly encouraged to allow extensive characterization prior to administration (FDA guidance documentation). We evaluated the impact of a second cycle of freeze/thaw on the cell viability at thawing, immediately before transplant into mice, and observed only a slight loss of viability upon thawing of Naive, GE- and LV-treated HSPCs (**Figure 4A**).

Next, to assess long-term gene correction *in vivo*, GE- and LV-treated CD34^+^ HSPCs from XSCID patients were transplanted *via* intra-hepatic injections into irradiated NSGS newborn pups for 16-18 weeks. The presence of circulating human cells was assessed by flow cytometry in the PB of transplanted mice at weeks 12 and 16-18 post-transplant (**Figure 4B**) and in the BM, spleen and thymus at 16-18 weeks (**Figure 4C**). The level of engraftment of GE XSCID HSPCs was comparable with HD and XSCID naive HSPCs. Except in the thymus, the human engraftment of LV-transduced HSPCs was lower than GE HSPCs (LV: 8.1±7.5% and GE: 23.3±13.9% hCD45^+^ cells in the bone marrow).

We also evaluated the percentages of human T, B and myeloid cell lineages in bone marrow following transplant (**Figure 4D**). We showed multilineage differentiation in the bone marrow of transplanted animals with presence of CD34^+^, CD3^+^ and CD19^+^ lymphoid and CD33^+^ myeloid cells. Impaired human T cell development in mice transplanted with Naïve XSCID HSPCs resembled human XSCID T-lymphopenia. However, the percentages of CD3^+^ T cells in GE-treated XSCID HSPCs animals (mean: 35.3%) were comparable with HD HSPCs (mean: 31.7%) (**Figure 4D**). GE consistently worked in achieving CD3+ T cell development in all four XSCID patient HSPCs, with significantly superior percentages in two of four patients (P2: 16.3% versus 0.6% and P3: 52.4% versus 2.8%, for GE and LV respectively), while LV worked in two of the four samples of which one resulted in a higher percentage than GE (P1: 54.5% versus 38% and P5: 85.1% versus 85.9%, for LV and GE respectively)(**Supplemental Figure S3A**). Percentages of CD3^+^ T cells in the PB, spleen and thymus of GE-treated XSCID HSPCs animals were also comparable with HD HSPCs transplanted animals (**Figure 4E**) as well as the ratio of CD4^+^, CD8^+^ and double positive (DP) T cell populations (**Supplemental Figure S3B**).

### *In vivo* maintenance of gene correction and functional correction

**Figure 5:**
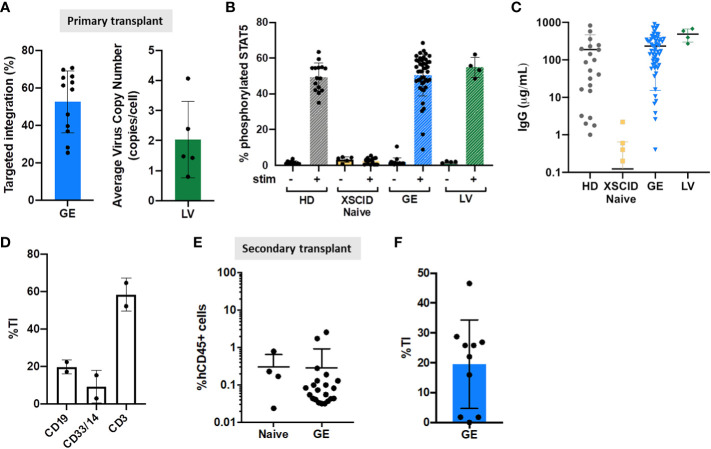
Genomic and functional correction in immunodeficient mice transplanted with GE- and LV-treated XSCID HSPCs **(A)** Quantification of TI rates and VCN by ddPCR in the human CD45^+^ cells isolated from the bone marrow of transplanted mice at week 16-18 post-transplant. **(B)** Phosphorylation of STAT5 was measured in hCD45^+^CD3^+^ T cells from the spleen by flow cytometry after 15 min stimulation with IL2/IL7 (+) or left unstimulated (-). **(C)** Human IgG production in immunodeficient mice transplanted with HD, XSCID naïve and GE- or LV-treated HSPCs cells. Concentration of IgG was quantified by ELISA in the serum of transplanted mice at week 16-18 post-transplant. **(D)** Quantification of TI rates B-lymphoid (CD19^+^), T-lymphoid (CD3^+^) and myeloid (CD33^+^/CD14^+^) cells after sorting from spleen of mice injected with GE XSCID HSPCs (n = 2). **(E)** Level of human CD45^+^ engraftment in mice bone marrow, at 16 weeks after secondary transplant with XSCID Naive (n = 4 mice) and GE-treated HSPCs (n = 21 mice) isolated from primary transplanted animals. **(F)** Quantification of TI rates in the human cells isolated from the bone marrow of secondary transplanted mice at week 16 post-transplant (n = 10 mice). Each symbol indicates a single animal.

To address potential problems with low engraftment of gene-edited HSPCs (26–28), we used a combination of gene editing enhancers (15). Here, we achieved an average of 52.57% TI in human cells isolated from the BM of GE HSPCs engrafted mice (**Figure 5A**), only slightly decreased compared to input cells (**Supplemental Figure S3B**, p=0.0137).

Signaling function of expressed IL2γc was assessed by STAT5 phosphorylation in CD3+ T cells following stimulation with IL2/IL7 (**Figure 5B**). We showed that pSTAT5 in splenic CD3^+^ T cells was restored in both XSCID GE- and LV-treated HSPCs to the same level as HD group (**Figure 5B**).

In addition to defective T cell immunity, B cells in XSCID are also defective as shown by impaired response to IL21. Therefore X-SCID patients requires life-long immunoglobulin supplement unless successfully transplanted. Our data confirmed IgG production in XSCID GE- and LV-treated HSPCs transplanted mice with serum IgG levels comparable to HD but no IgG detected in Naive XSCID HSPC mice (**Figure 5C**).

Sorting of B-lymphoid (CD19^+^), T-lymphoid (CD3^+^) and myeloid (CD33^+^/CD14^+^) cells from spleen of GE XSCID HSPCs injected mice confirmed the high level of gene correction in T cells (mean TI 58.4%), and to a less extent the correction of B-lymphoid and myeloid cells (**Figure 5D**).

Despite a low level of human engraftment in a secondary transplant from GE XSCID HSPCs mice (**Figure 5E**), average TI was of 19.54% (range 1.79-46.58%) from BM (**Figure 5F**).

### Safety assessment after gene editing of XSCID HSPCs

To perform an extensive off-target analysis, we applied rhAmpSeq (IDT) to evaluate 82 potential off-targets identified by CHANGE-seq and 1 on-target. We included in the assessment *in vitro* (3 samples) and *in vivo* (3 samples) samples harvested from transplanted mice. No significant off targets were observed above the background sequencing noise of ~2-3%. Two variants at OT8 and OT64 that were detected are single nucleotide polymorphisms in the patient that differed from the reference genome, unrelated to CRISPR/Cas9 editing.

**Figure 6:**
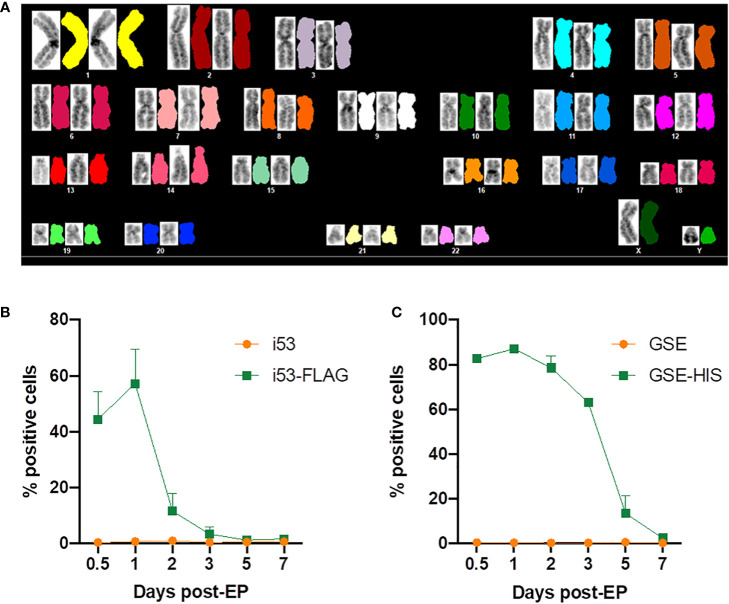
Evaluation of genomic integrity and persistence of gene editing enhancers. **(A)** Representative spectral karyotype image (12 metaphases imaged) of GE XSCID HSPCs analyzed using HiSKY. **(B)** Percentage of cells expressing i53 protein detected using an antibody directed against the FLAG tag and measured by flow cytometry at the indicated days post-electroporation (EP) (n = 2 independent experiments). **(C)** Percentage of cells expressing GSE CS-56 protein detected using an antibody directed against the HIS tag and measured by flow cytometry at the indicated days post-EP (n = 2 independent experiments).

Spectral karyotyping was performed to assess large chromosomal changes such as deletions and translocations. Twelve metaphases analyzed showed normal chromosomal content and arrangement within the limit of its detection (**Figure 6A** **).**

In addition, the kinetics of expression of i53 and GSE CS-56 proteins after electroporation was assessed by flow cytometric analysis. Since there is no commercial antibody available, mRNAs encoding for a tagged version of i53 (i53-FLAG) and GSE CS-56 (GSE-HIS) were produced and used during gene editing. We showed that i53 protein expression peaks at 1 day post-electroporation, with 60% positive cells, then decreased rapidly with approximately 10% cells expressing i53 at day 2, time of freezing for future transplant (**Figure 6B**). GSE CS-56 protein expression also peaked at 1 day post-electroporation with >80% positive cells (**Figure 6C**). However, the decrease of expression was slower with 7-20% GSE CS-56-expressing cells still present at day 5 post-GE.

**Figure 7:**
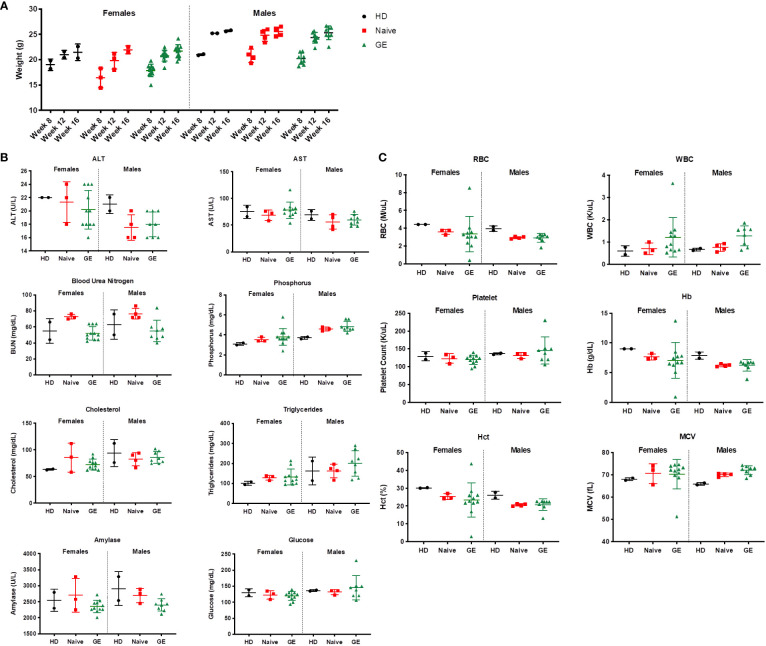
Impact of engraftment with GE HSPCs on weight, CBC and chemistry parameters **(A)** Weight (g) of mice was measured at indicated time points during the safety studies and reported for female and male animals transplanted with HD or with XSCID Naïve or GE HSPCs. **(B)** Serum from transplanted animals was analyzed at week 16 post-transplant for the different chemistry parameters as indicated on top of each graph. **(C)** Whole blood was analyzed at week 17 post-transplant for the different hematology parameters as indicated on top of each graph. Each symbol indicates a single animal.

In addition, preclinical safety studies were performed in transplanted mice to detect potential toxicities after engraftment of gene edited HSPCs (**Figure 7**). During this study, the mortality rate for mice injected with Naive XSCID HSPCs (3.58%, n = 28 mice, 1 death) is comparable with mice injected with GE XSCID HSPCs (4.26%, n = 94 mice, 4 deaths). Weights were also similar between animals injected with HSPCs HD and XSCID Naive or GE (**Figure 7A**). Data from chemistry (**Figure 7B**) and hematology (**Figure 7C**) parameters were within reported ranges for humanized immunodeficient mice (29) and consistent between the different conditions (HD, XSCID Naive or GE). In addition, necropsy of animals detected no signs of tumor/malignancy.

## Discussion

A key rationale for *ex vivo* gene therapy with the CRISPR-Cas9 targeted *IL2RG* insertion approach compared to current lentiviral vector-integrated *IL2RG* is the precise insertion of *IL2RG* at the endogenous locus to achieve physiological *IL2RG* expression and function, and abrogate most off-target genomic alterations.

Lentivectors can effectively insert genes of interest into relatively quiescent HSPCs. Of note, the vector copy number from pooled cells may over-estimate the actual percentages of cells corrected because some clones may harbor multiple copies of the transgene per cell. Vector copy number in colony forming units (CFU)s derived from CD34^+^ HSPC in the first three older XSCID patients who achieved T- and B-cell restoration ranged from 6% to 10%, suggesting this frequency of corrected HSPCs is sufficient to restore T- and B-cell immunity (7). Recent development in adding transduction enhancers during lentivector transduction with use of LentiBoost and/or prostaglandin E2 has greatly increased total VCN (30–32), including clones with multiple vector inserts per cell, and whether the increased VCN lead to improved clinical outcomes remain unknown.

In contrast, unlike the possibility to harbor multiple copies of LV-*IL2RG*, on-target TI of *IL2RG* at the endogenous locus in principle cannot exceed one insertion per cell in a male individual with only one X-target. Therefore, the comparison is between multi-copy weaker-expressing *IL2RG* from LV versus lower frequency but endogenously regulated *IL2RG*.

Functionally, the level of *IL2RG* correction is also reflected by the efficiency at which gene corrected XSCID CD34^+^ HSPCs develop and differentiate into functioning lymphocytes. Upon IL2 stimulation, γc binds with IL2Rβ and α chain to form a high affinity receptor for IL2 and triggers phosphorylation of Jak3 and downstream STAT5 (33). We showed normalized phospho-STAT5 signaling in animals that received TI-corrected HSPCs as least as effective as mice transplanted with LV-HSPCs. Consistent with the critical role γc plays in T cell differentiation, *IL2RG*-defective HSPCs exhibit an early block in T cell differentiation. The recently described ATO model has been instrumental in uncovering lymphocyte differentiation blocks in multiple IEIs (18, 34). Here, we demonstrated the block at Pro-T stage in naïve XSCID HSPCs relieved and both LV- and GE-*IL2RG* HSPCs differentiating to Pre-T-I and Pre-T-II cells (33.1% and 35.3% for LV- and GE-corrected cells respectively).

We transplanted corrected CD34^+^ HSPCs intra-hepatically into NSGS mice shown previously to support lymphocyte development (12). Data show higher percentages of CD3^+^ T lymphocytes in NSGS peripheral blood and spleen when transplanted with GE-*IL2RG* HSPCs. Importantly, peripheral blood lymphocytes from transplanted mice transplanted with either GE- or LV-corrected XSCID HSPCs demonstrated comparable STAT5 phosphorylation upon stimulation.

Natural killer cells are of special interest, the development of which following gene therapy is poorly understood. There are conflicting reports of the level of γc expression necessary for NK development (35). The LV VCN in peripheral blood NK cells increase over time (7), suggesting an outgrowth of NK cells with higher copies of transgene. *In vitro* NK differentiation of GE-*IL2RG* HSPCs was significantly superior than LV-corrected cells (40.7% versus 2.8% LV-corrected); however, this was surprisingly not observed in transplanted mice based on the percentages of CD3^-^CD56^+^ cells in CD45^+^-gated population. Of note, the majority of adult human XSCID subjects treated with lentivector gene therapy did not achieve normal NK reconstitution (7) despite high vector copy numbers, raising suboptimal γc expression as a potential contributing factor. The timing of the exposure to various cytokines may be important in γc-mediated signaling and proper NK cell development and maturation.

The ideal approach for genetic modification to restore cellular function depends on the disease. Lack-of-function diseases that are not highly regulated may do well with over-expression from a small percentage of the cells for example, metachromatic leukodystrophy, or adenosine deaminase deficiency (36). In contrast, highly regulated diseases require physiological regulation by an endogenous promoter as previously shown in CD40L XHIM disease (37). In patients with XSCID, restoring cellular regulation by the endogenous promoter may provide optimal protein expression necessary for lymphocyte development and immune cell interactions.

An important consideration when comparing LV and CRISPR-Cas9-TI approaches at *IL2RG* is safety. Semi-random LV-*IL2RG* insertions and the consequent safety concerns are well described, particularly with strong enhancers as reported in the lentiviral gene therapy for cerebral adrenoleukodystrophy (Servick K. Gene therapy clinical trial halted as cancer risk surfaces. Science News. 2021. Available at: https://www.science.org/content/article/gene-therapy-clinical-trial-halted-cancer-risk-surfaces). Preferential LV insertion in coding regions heightens risk for aberrant fusion transcripts between vector and insertion gene have resulted in clonal HMGA2 expansions in β-Thalassemia and XSCID (10, 11). Efforts to reduce the effects of ectopic expression include the use of myeloid-specific promoters (38, 39) or the inclusion of endogenous regulatory elements in the vector (40). The EF1A promoter in the clinical LV for XSCID was chosen for its relatively weak expression in response to the leukemic events in prior gRV gene therapy for XSCID. Whether stronger and physiologically regulated γc expression might accomplish a more robust and expedient immune recovery remains to be answered and may be provided by treatment of XSCID patients with a targeted *IL2RG* insertion approach.

The rates of off-target cleavage are highly dependent on the guide sequence, and as shown by Pavel-Dinu et al. (12) and our data, a mere shortening the guide by one base pair can substantially reduce or eliminate off-targets observed with sgRNAs bearing canonical 20 bp spacers. Our inability to detect any OTs either before or after transplant using rhAmpseq, a highly sensitive NGS-based assay, across 82 OTs is reassuring that the specificity of cleavage is restricted to the on-target site, at least within the limits of state-of-the-art assays.

Exposure to AAV, more so than double strand DNA breaks, can cause significant DNA damage response (15, 41, 42) that likely contributed to the significant decline in engrafting GE HSPCs observed previously. A p53 inhibitor delivered as mRNA to suppress this response significantly improves the survival of engrafting gene-edited HSPCs by acutely interrupting the chain of reactions of cell cycle arrest, senescence and apoptosis (12, 13). Reports of deleterious effects of p53 pathway suppression and tumorigenicity are associated with long-term p53 blockade, unlike the transient effect provided by the GSE CS-56 mRNA in this report. The short-lived action as shown by our data confirms the effect is short-lived, and did not increase indels at OT sites *in vitro* or following transplant. Normal karyotype studies and the absence of tumors in transplanted animals are reassuring, although lack sensitivity and have not shown to be predictive of tumors in previous *ex vivo* gene therapy clinical trials.

In conclusion, our comparison of *IL2RG* gene correction approaches *via* integrating lentivector or CRISPR-Cas9 TI demonstrates that it is possible to achieve comparable levels of gene correction. The rates of off-target indels induced by CRISPR-Cas9 are almost non-existent, suggesting a more predictable and less genotoxic safety profile compared to randomly integrating LV. Our data shows targeted gene editing as an efficient and safe approach to control the insertion of corrective cDNAs at endogenous loci, benefitting from physiological regulation of the target gene. Absence of toxicity associated with gene editing in our preclinical studies further supports a Phase I/II clinical trial to allow longer term efficacy assessment for immune reconstitution, particularly in NK cells and restoration of humoral immunity.

## Data availability statement

The original contributions presented in the study are included in the article/**Supplementary Materials**. Further inquiries can be directed to the corresponding author.

## Ethics statement

The animal study was reviewed and approved by NIAID Institutional Animal Care and Use Committee.

## Author contributions

JB, SD conceived and performed most of the experiments and wrote the manuscript. TL assisted with functional studies. TL, AL, NK, ME, NK, VB helped with mice experiments. ME performed mouse pathology. SL, XW, SB performed genomic analysis. CL, ST performed and analyzed CHANGE-seq assay. MB, LN provided advices to perform the ATO system. UC, MP-D designed the rAAV6 donor. RM designed IVT templates and mRNA constructs. SB performed karyotyping and FISH studies. BK, LN, GD, MP, HM provided expert advice and guidance and assisted with writing the paper. All authors contributed to the article and approved the submitted version.
