# Supplementary figures and images for: CRISPR-Cas9-AAV versus lentivector transduction for genome modification of X-linked severe combined immunodeficiency hematopoietic stem cells
Source: Front Immunol. 2023 Jan 4;13:1067417. doi: 10.3389/fimmu.2022.1067417 (PMC9846165; doi:10.3389/fimmu.2022.1067417)

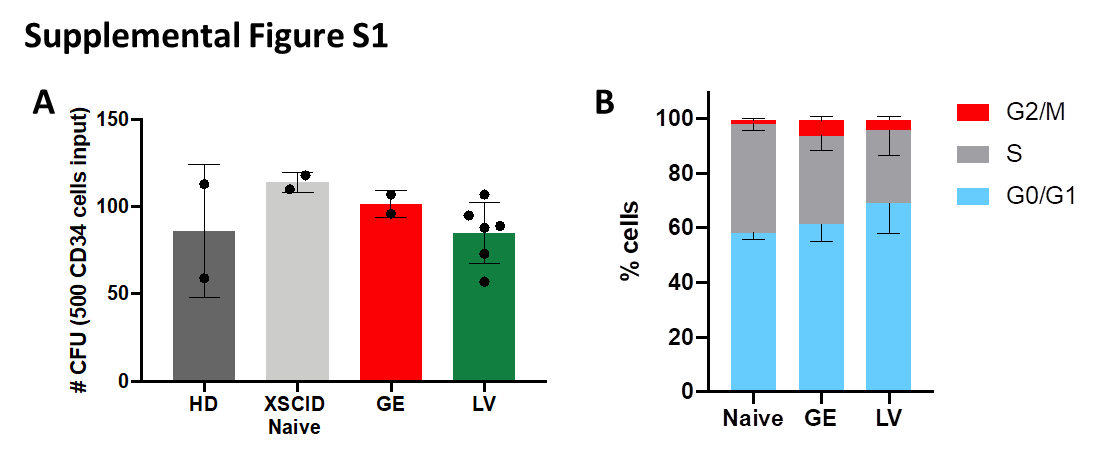

Supplement: Supplementary file 1 [file Image_1.tif]

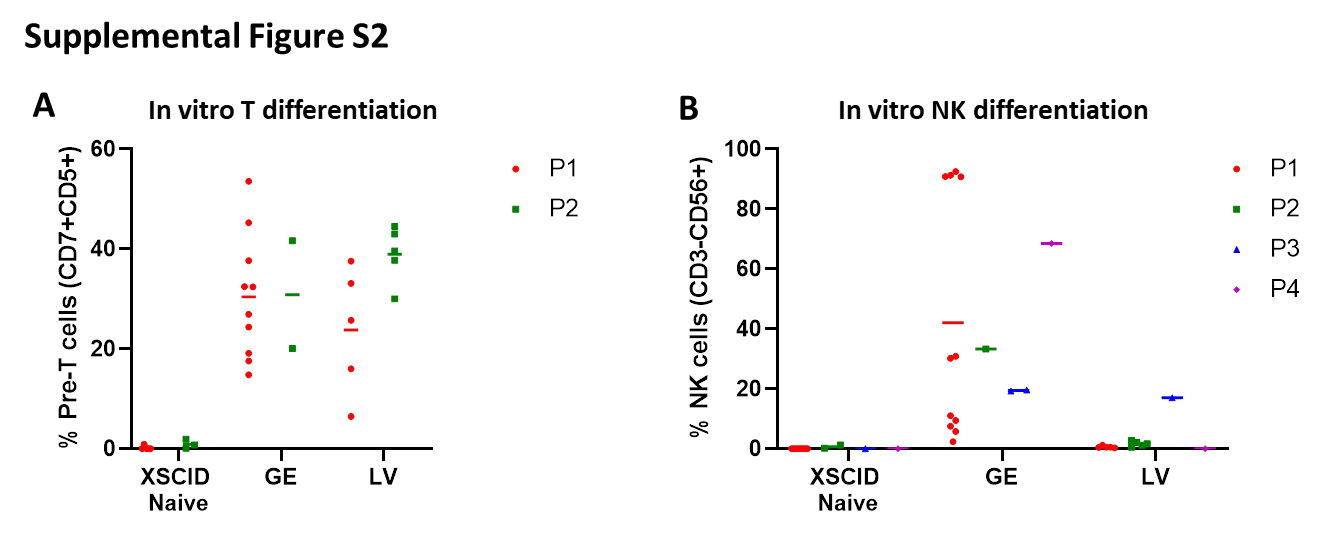

Supplement: Supplementary file 2 [file Image_2.tif]

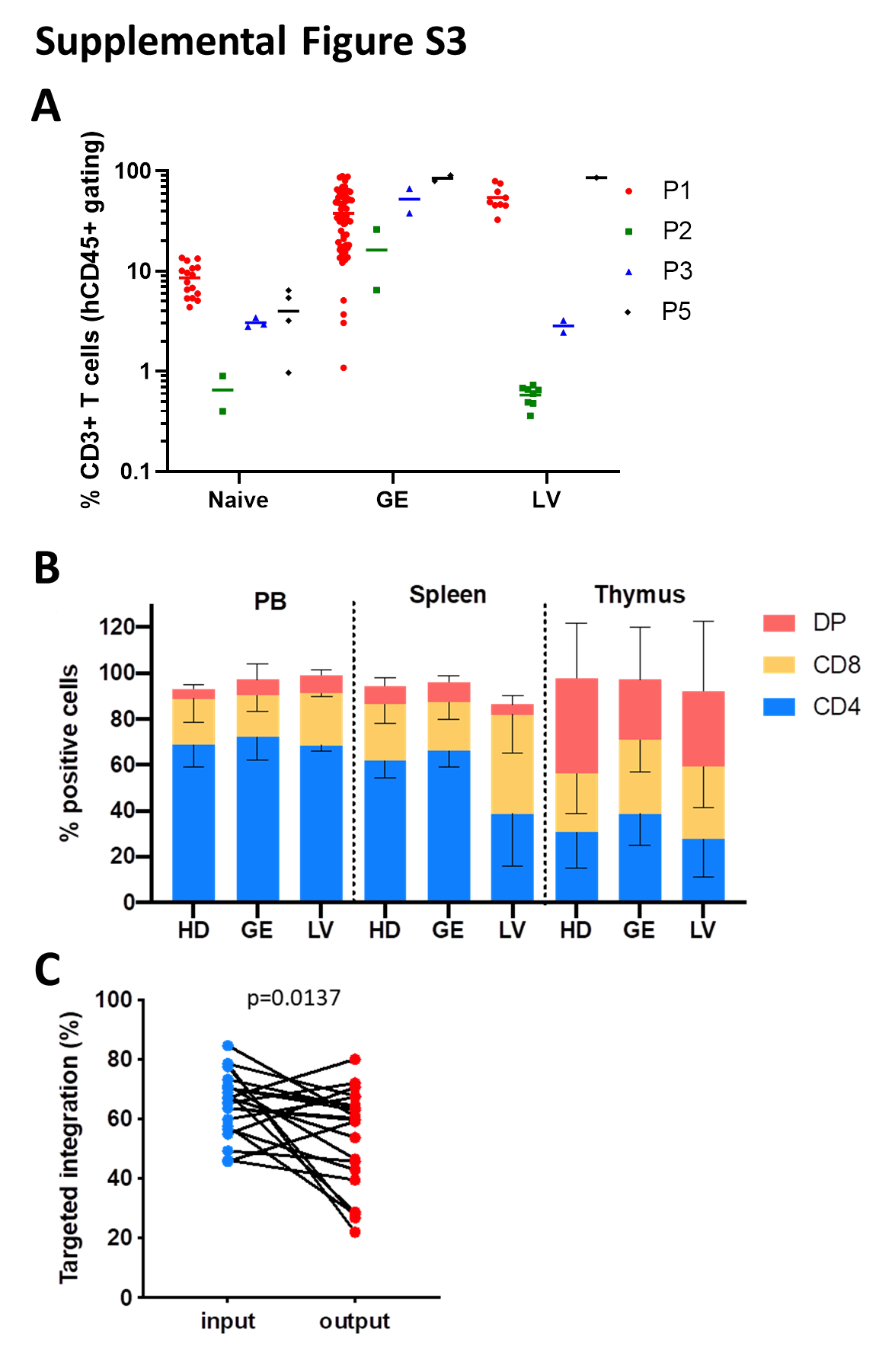

Supplement: Supplementary file 3 [file Image_3.tif]
